# Risk-Based Control Strategies of Recombinant Monoclonal Antibody Charge Variants

**DOI:** 10.3390/antib11040073

**Published:** 2022-11-20

**Authors:** Alain Beck, Christine Nowak, Deborah Meshulam, Kristina Reynolds, David Chen, Dennis B. Pacardo, Samantha B. Nicholls, Gregory J. Carven, Zhenyu Gu, Jing Fang, Dongdong Wang, Amit Katiyar, Tao Xiang, Hongcheng Liu

**Affiliations:** 1 Centre d’Immunologie Pierre-Fabre (CIPF), 5 Avenue Napoléon III, 74160 Saint-Julien-en-Genevois, France; 2 Protein Characterization, Alexion AstraZeneca Rare Disease, 100 College St., New Haven, CT 06510, USA; 3 Technical Operations/CMC, Scholar Rock, 301 Binney Street, 3rd Floor, Cambridge, MA 02142, USA; 4 Protein Sciences, Scholar Rock, 301 Binney Street, 3rd Floor, Cambridge, MA 02142, USA; 5 Research, Scholar Rock, 301 Binney Street, 3rd Floor, Cambridge, MA 02142, USA; 6 Jasper Therapeutics, Inc., 2200 Bridge Pkwy Suite 102, Redwood City, CA 94065, USA; 7 Biological Drug Discovery, Biogen, 225 Binney St., Cambridge, MA 02142, USA; 8 Global Biologics, Takeda Pharmaceuticals, 300 Shire Way, Lexington, MA 02421, USA; 9 CMC Technical Operations, Magenta Therapeutics, 100 Technology Square, Cambridge, MA 02139, USA; 10 Downstream Process and Analytical Development, Boston Institute of Biotechnology, 225 Turnpike Rd., Southborough, MA 01772, USA

**Keywords:** charge variants, comparability, critical quality attributes, monoclonal antibodies, specification

## Abstract

Since the first approval of the anti-CD3 recombinant monoclonal antibody (mAb), muromonab-CD3, a mouse antibody for the prevention of transplant rejection, by the US Food and Drug Administration (FDA) in 1986, mAb therapeutics have become increasingly important to medical care. A wealth of information about mAbs regarding their structure, stability, post-translation modifications, and the relationship between modification and function has been reported. Yet, substantial resources are still required throughout development and commercialization to have appropriate control strategies to maintain consistent product quality, safety, and efficacy. A typical feature of mAbs is charge heterogeneity, which stems from a variety of modifications, including modifications that are common to many mAbs or unique to a specific molecule or process. Charge heterogeneity is highly sensitive to process changes and thus a good indicator of a robust process. It is a high-risk quality attribute that could potentially fail the specification and comparability required for batch disposition. Failure to meet product specifications or comparability can substantially affect clinical development timelines. To mitigate these risks, the general rule is to maintain a comparable charge profile when process changes are inevitably introduced during development and even after commercialization. Otherwise, new peaks or varied levels of acidic and basic species must be justified based on scientific knowledge and clinical experience for a specific molecule. Here, we summarize the current understanding of mAb charge variants and outline risk-based control strategies to support process development and ultimately commercialization.

## 1. Introduction

Typical recombinant monoclonal antibodies (mAbs) are assembled with two identical light chains and two identical heavy chains that are connected by inter-chain disulfide bonds to form a tetrameric structure. Each light chain is encoded by a single light chain gene and each heavy chain is encoded by a single heavy chain gene with the expectation of a uniform molecule. However, heterogeneity is introduced throughout the lifespan of mAbs, stemming from low-frequency errors that occur during DNA replication and transcription, amino acid misincorporation, structural variation from folding and solution dynamics, posttranslational modifications, and degradations. The heterogeneity is reflected in differences in molecular weights, hydrophobicity, and more commonly, charge, to generate the so-called charge variants. This heterogeneity was first demonstrated experimentally using a plasma tumor cell line expressing an IgG2 molecule, where a single band was observed by isoelectric focusing (IEF) gel electrophoresis within ten minutes of synthesis but was converted into multiple bands of different isoelectric points (pI) within an hour [1]. Charge heterogeneity has attracted substantial attention throughout mAb therapeutic development because charge profiles are sensitive to process changes and the resulting differences need to be justified to have no adverse effect on safety and efficacy. Various modifications causing charge heterogeneity have been identified. Variation of charge variants with process parameters during cell culture, purification, and formulation development has been widely studied. Such knowledge has been used to design appropriate control strategies for maintaining consistent charge profiles throughout development and commercialization to mitigate safety and efficacy risks. Beyond process controls, developability optimizations made prior to lead candidate selection, such as the design of display libraries that are free from common sequence-based liabilities, have become a growing industry trend that can improve stability, pharmacokinetics (PK), and pharmacodynamics (PD) [2,3].

A typical charge profile of an mAb contains one major and several minor species when analyzed by ion exchange chromatography (IEX) or IEF- based methods. They are defined as acidic, main, and basic species. Species with relatively lower pI than the major species are referred to as acidic species, while species with relatively higher pI are referred to as basic species. A wide range of acidic species and basic species have been observed in mAbs approved by the FDA and the European Medicines Agency (EMA) [4,5], reflecting the diversity of molecules and processes that can result in different types and different levels of modifications.

Acidic and basic species are not of concern for mAb development if these species are controlled within the acceptable range of clinical activity. These species will be considered clinically qualified for safety and efficacy. However, variation outside of this range requires careful evaluation of the impact on product safety and efficacy based on the nature of modifications, structure–function relationship, and the phase of development. For example, the presence of a C-terminal lysine (Lys) is the major factor causing basic species, but variation in this attribute should be considered as low risk because it has no effect on mAb structure, stability, or potency, and can be removed rapidly in circulation in humans [6]. On the other hand, asparagine (Asn) deamidation in complementarity-determining regions (CDRs), a major source of acidic species, needs to be carefully monitored and controlled because it can decrease potency [7]. It has been suspected that substantial pI differences can affect mAb biodistribution and PK [8]. However, experimental data from studies using animal models with separated acidic and basic species revealed minimal PK differences between acidic and basic species from the main peak either by intravenous or subcutaneous injections [9,10]. These data suggest that the differences in pI and other quality attributes between acidic, main, and basic species are not sufficient to cause measurable differences in the measured attributes including PK. However, caution should be taken before generalizing such a conclusion because a specific modification when highly enriched in acidic or basic species could cause a difference in potency, such as Asn deamidation in CDRs [7]. Additionally, for a specific mAb, the presence of unique modifications cannot be excluded without extended characterization of the isolated peaks. Therefore, without a detailed characterization of acidic and basic species, it is an appropriate strategy to maintain a consistent charge profile. As an exception to decreased PK caused by pI difference, substantial levels of methionine (Met) oxidation have been shown to cause a shorter half-life, while it has no impact on pI [11,12].

From the control strategy perspective, it is beneficial to understand the mechanisms for various modifications, such as enzymatic or non-enzymatic reactions and dependence on pH and temperature. The charge profiles of mAbs from bioreactors can be replicated by in vitro incubation [13,14,15], suggesting that most of the modifications are generated by exposure to environmental factors. Studies further demonstrated that deamidation of the same susceptible Asn residues followed similar kinetics in vitro or in vivo in mice [16], cynomolgus monkeys [17,18], or humans [19]. The similarity between in vitro and in vivo modifications has also been observed for glycation [20], thioether formation [21], and formation of pyroglutamate (pyroGlu) from N-terminal glutamate (Glu) [22]. Many modifications that are identified in mAbs can also be found in endogenous human IgGs or other human proteins [16,19,20,21,22,23,24,25]. The generation of similar charge variants from the manufacturing process, in vitro incubation, and in vivo in animals and humans provides a good justification to alleviate the development risks from the safety perspective.

Acidic and basic species are sensitive to process changes, indicating the need for careful consideration throughout process development including scale-ups, transferring to different manufacturers, process optimization, and commercialization. However, the lack of a full understating of the chemical nature of acidic species and basic species and the process’s impact on charge heterogeneity poses one of the highest risks to establishing comparability for successful development from early to late and commercial stages. This review summarizes the current understanding of mAb acidic and basic species and provides a scientific foundation to evaluate acidic and basic species during development. It provides a practical framework for risk assessment of critical quality attributes (CQA) and control strategies, including upstream, downstream, stability, and setting phase-appropriate specifications.

## 2. Modifications of Acidic and Basic Species

Commonly, acidic species are detected as several small peaks when analyzed by IEF- or IEX-based techniques, formed due to modifications such as deamidation, glycation, and sialyation. In contrast, basic species are usually detected as fewer peaks, formed due to fewer modifications such as in-complete removal of C-terminal Lys or C-terminal amidation.

### 2.1. Modifications in Acidic Species

Modifications contributing to the formation of acidic species are listed in Table 1, classified based on the prevalence. Common modifications are a reflection of the general properties of mAbs and processes such as cell line, cell culture, and purification. On the other hand, rare modifications are more likely due to unique mAbs’ properties and unique cell culture or environmental factors. These modifications occur mainly during cell culture, and to a lesser degree, during purification and storage. Several modifications cause the formation of acidic species by directly altering the number of charges, either by adding negative charges, such as deamidation or by reducing the number of positive charges such as glycation of Lys residues. Interestingly, modifications enriched in acidic species without direct effect on charges have been commonly observed when analyzed by IEX methods.

Asn deamidation, sialylation, and glycation are almost ubiquitously enriched in acidic species. Deamidation and sialylation cause the formation of acidic species by adding negative charges, while glycation causes the formation of acidic species by masking the positive charges. A typical mAb contains around 50 Asn residues that are potentially susceptible to deamidation when located in the appropriate structure context and exposed to environmental factors such as slightly basic pH and elevated temperatures. Asn deamidation converts Asn into negatively charged isoAsp and Asp. Both isoAsp and Asp are more acidic than the original Asn, however, there are subtle differences between the two. MAbs containing isoAsp have been found in either basic [26,28,34] or acidic [32] fractions when analyzed by chromatography methods, suggesting additional separation mechanisms of chromatography methods. Low levels of sialylation are expected due to the presence of substantial amounts of N-linked glycans containing terminal galactose as a substrate and the presence of the enzymatic machinery in mammalian cell lines [9,27,30,36,39,43,46]. MAbs with glycosylation in the fragment antigen binding (Fab) region could accommodate higher levels of sialic acid [4], thus contributing more significantly to acidic species. Glycation is a non-enzymatic reaction between amine groups and reducing sugars. It is a common factor in the formation of acidic species [9,14,29,34,36,37,39,47,48] due to the large number of Lys residues in mAbs and sugars used as nutrients in cell culture.

Often, acidic species separated by IEX methods may contain modifications that do not correlate with their charge properties. It has been hypothesized that those modifications cause conformational changes leading to surface charge redistribution and thus differences in interactions with column resins [56]. As shown in several cases, the same modifications can cause the formation of either acidic or basic species. Succinimide, as an Asp isomerization intermediate, is predicted and has been confirmed experimentally, to contribute to the formation of basic species because of the loss of a negative charge compared to Asp [26,57,58,59]. However, succinimide has also been shown to be enriched in an acidic species [34]. Oxidation without direct impact on charges has been reported to be enriched in either acidic [14,29] or basic species [36,60,61]. With the minimal difference in the side chain pKa between Asp and isoAsp, mAbs with isoAsp have been found in either acidic [32] or basic [26,28,34] species or coeluting with mAbs containing Asp [59]. Similarly, mAbs with the unformed heavy chain variable domain disulfide bond have been found to elute with acidic species in one study [49] and with basic species in another study [62]. For IgG2, the different inter-chain disulfide bond linkage leads to the formation of IgG2 isoforms, the classical IgG2A, later discovered IgG2B, and the intermediate IgG2A/B. These have been shown to impact the chromatographic separation of mAbs, e.g., IgG2A is more acidic [32,50] or more basic [24,34] than IgG2A/B and IgG2B, reflecting the sensitivity of IEX chromatography or disulfide bond isoforms affecting charge distribution in a molecule-specific manner. Cysteine (Cys) residue-related modifications including non-reducible species [9] trisulfide bonds [32,50] and cysteinylation and glutathionylation [39] probably also cause the formation of acidic species through structural modulation. Fragments have also been found commonly in acidic species [33,39,45], caused either directly by charge differences or indirectly by altering structures and thus interactions with column resins. The lack of a direct correlation between charges and chromatography separation highlights the importance of the characterization of the separated peaks.

In addition to these common modifications, there are several modifications that have been reported in only one or two cases. Modification of a mAb by tyrosine sulfation was discovered in the fraction that is tightly bound to an anion exchange column operating under the flow-through mode for impurity clearance [52]. This modification can be easily missed from the analysis of the bulk drug substance since it can be completely removed by this chromatography step. Tyrosine sulfation requires tyrosylprotein sulfotransferase and the specific structural motif present in the mAb to occur [52], which may explain why it was not widely reported. Another example is a sequence variant with a glycine (Gly) substituted by an Asp [29] and leader sequences with acidic amino acids [38] are enriched in acidic species due to the addition of negative charges to mAbs. Both sequence variants and the presence of leader sequence are commonly detected in mAbs. However, it is possible that they are either completely cleared by purification steps or present at extremely low levels in bulk drug substance and are thus not being reported as a common factor in acidic species. Maleuric acid derivatives of the light chain and heavy chain N-termini were detected in a mAb that is expressed in transgenic goats and secreted in milk [51] and thus it is highly doubtful that the same modification can be found in mAbs expressed in cell lines. Modifications of arginine residues by methylglyoxal were found in a mAb expressed in a CHO cell line using a chemically defined medium [55] and this modification caused the formation of acidic species by masking positive charges. Modifications by citric acid caused the formation of acidic species by direct contribution to negative charges and masking positive charges [53]. Aggregates, as will be discussed in the following section, have been commonly found in basic species, but they can also be found in acidic fractions [33]. As shown by this study, caution should be taken when analyzing aggregates due to potential sample handling artifacts [33]. Naturally, during fraction collection and concentration, the acidic and basic species are of low abundance and subject to more stress and thus are highly likely to introduce artifacts, e.g., procedure-induced aggregate.

Several studies have demonstrated that the identified modifications can fully account for acidic species. For example, Perkins et al. [13] studied the charge variants of a murine monoclonal IgG1 antibody using a strong cation exchange column, where three major groups of peaks with each group further containing three peaks were observed. It was determined that the peak pattern was caused by various combinations of deamidation on the light or heavy chains. Harris et al. [26] reported an in-depth characterization of the charge variants of trastuzumab, where three modifications were identified in the acidic peaks, including deamidation of Asn30 in the light chain CDR1, deamidation of Asn55 in the heavy chain CDR2 and isomerization of Asp102 in the heavy chain CDR3. The observed acidic peaks can be fully accounted for by the three observed modifications. Acidic species have also been fully accounted for by the identified modifications in several additional studies [9,15,27]. A full accounting of acidic species by the identified modifications eliminates uncertainty for risk assessment of the acidic species.

In other studies, acidic species could not be fully accounted for by the identified modifications [30,32,38,39]. Tang et al. [30] characterized the acidic species of an IgG1 mAb expressed in NS0 cells, where 19.6% deamidation in the “PENNY” (single letter coded amino acid sequence) peptide and 14.5% sialylation were identified. Considering the dimeric structures, deamidation and sialylation can account for approximately 60% of the identified acidic species. Neill et al. [32] used a combination of OFFGEL fractionator and anion exchange chromatography to enrich acidic and basic fractions of a recombinant monoclonal IgG2/4 hybrid molecule. They observed a slightly higher level of deamidation in the “PENNY” peptide and isoAsp from the isomerization of Asp, and a slightly higher level of trisulfide bond. However, the three identified modifications can only account for approximately 20% of the total acidic species. When acidic species cannot be fully accounted for, there is an additional risk due to uncertainty regarding the nature of modifications and their impact on safety and efficacy, which makes risk assessment challenging.

### 2.2. Modifications in Basic Species

Various modifications that have been identified in basic species are summarized in Table 2, classified based on the prevalence. In contrast to acidic species, basic species can be fully accounted for by a few identified modifications arising mainly from cell culture, and to a lesser degree from purification and storage.

Several common modifications generate basic species by adding positive charges. The most common modification causing basic species is C-terminal Lys. Since C-terminal Lys has no significant impact on mAb functionality, one common approach to reduce heterogeneity is to remove the codon for heavy chain C-terminal Lys. C-terminal amidation is also a common factor contributing to the formation of basic species. Both C-terminal Lys removal and C-terminal amidation are catalyzed by enzymatic reactions; therefore, their levels are influenced by cell culture conditions. MAbs with N-terminal glutamine (Gln) are enriched in the basic species [27,62,64,65] because cyclization of N-terminal Gln to pyroGlu causes a loss of positive charges. Other common modifications that have been identified in basic species include the presence of partial leader sequence [9,27,32,34,38,45,64,69], succinimide as intermediate for both Asp isomerization [26,57,58,59] or Asn deamidation [44] intermediates, and aggregates [9,10,33]. Using ion-exchange columns in tandem with the size-exclusion column, it was found that aggregates are eluted in the late fractions from both cation and anion-exchange columns [72]. The authors hypothesized that the later elution could be due to secondary hydrophobic interactions between aggregates and column resins. While it is also possible that the later elution is simply due to the increased numbers of both negative and positive aggregates. In a few cases, modifications such as sequence variants [70,71], unformed disulfide bonds, [33,62] cysteinylation [38], and N-terminal Glu cyclization [39,69] have also been found in basic species.

Basic species observed by chromatography-based methods can also be generated by modifications without direct impact on charges. Free Cys residues from unformed heavy chain variable domain disulfide bonds were found in basic species in one study [62], while it was also found in acidic species in another study [49]. MAbs with Asp isomerization have been found in basic species [26,34,42], rarely in acidic species [32], or co-eluting with the original Asp [59]. Several studies have demonstrated that oxidation of Met caused the formation of basic species [36,46,60,61]. The elution order of IgG2 disulfide bond isoforms has also been shown to vary in different studies [24,32,34,50]. Additionally, cysteinylation can also cause the formation of basic species [38]. Those examples, such as the separation of acidic species, indicate the sensitivity of chromatography methods to subtle changes in mAb conformation and highlight the need for direct characterization of isolated fractions.

## 3. Impact of Acidic and Basic Species on mAb Structure, Stability, and Biological Activity

For early phase programs, it is a balance between rapidly advancing programs to Investigational New Drug (IND)-enabling toxicology followed by first-in-human studies while still gaining sufficient process understanding. It is thus challenging to maintain acidic and basic species within a reasonable range that does not impact safety and efficacy when advancing programs to late-stage with inevitable process improvement, transfer, and manufacturing scale-up. For biologics license applications (BLA), acidic and basic species are required to be thoroughly evaluated using separated fractions. To establish the direct link between a specific modification and its impact on structure and functionality, material enriched with one modification are required. Such materials can either be separated using preparative IEF or chromatography methods. If separation is not feasible due to peak overlap, forced degradation conditions can be used to generate materials with the target modifications, such as oxidation with hydrogen peroxide, and deamidation with high pH and high temperature. Forced degradation conditions should be carefully selected to mainly modify the same sites that are present in those identified in the separated acidic and basic species. When modifications at other sites cannot be avoided, the impact of off-target modifications should be considered.

The impact of Asn deamidation has been extensively studied due to its ubiquitous presence in acidic species. Deamidation has been localized mainly to CDRs [15,16,17,18,26,28,37,44,73,74] and the “PENNY” peptide in the Fc region [9,16,27,31,32,33,36,37,39,75,76,77]. To a much lesser degree, deamidation has been detected in other sites [9,13,31,36,38,40,75,78]. Asn deamidation in CDRs has been demonstrated, with almost no exception, to cause decreased antigen binding [15,16,17,18,26,28,37,44,74,79] with no [16] or minimal [28] effect on conformation. From the developability perspective, Asn in CDRs, especially when followed by Gly or serine making it highly prone to deamidation should be carefully evaluated by forced degradation studies to determine the propensity for deamidation. Deamidation in the “PENNY” peptide did not alter mAbs’ conformations [9,30,80], but it has been shown to slightly decrease FcRn binding [9] and increase aggregation including the formation of particulates at both low and neutral pH [81]. Other than aggregation, deamidation in the “PENNY” peptide may not be a significant concern, but its level should be controlled due to its impact on charge heterogeneity, which is routinely monitored by batch release and stability to demonstrate process consistency and comparability.

Although the presence of sialic acid is not a concern for the development of mAbs due to its low percentage, the impact of sialic acid on IgG structure, stability, and biological activities has been widely studied, at times producing conflicting results. Sialic acid has been shown to cause a subtle conformational change around the glycosylation sites [82,83], without any global impact on secondary and tertiary structure [9], nor unfolding stability [30]. Multiple studies have demonstrated that sialic acid has no impact on antigen binding [9,27,84,85], complement-dependent cytotoxicity (CDC) [82,85], antibody-dependent cellular cytotoxicity (ADCC) [82,85] or in vivo half-life [9,82,84]. In contrast, several studies demonstrated that sialic acid may cause slightly decreased binding to FcRn [9], and decreased ADCC [84,86]. One potential contributing factor to the conflicting results on ADCC is probably differences in the degree of sialylation, e.g., lower levels of sialylation causing no detectable difference in IgG’s interactions with Fc receptors. Additionally, the decreased ADCC in one of the studies was attributed to decreased binding to FcγIIIa on natural killer cells and lower binding affinity to cell surface antigen caused by repulsion between the negatively charged cell surfaces and sialic acid [86].

Glycation is another common modification mainly arising from cell culture because of the use of sugars as essential nutrients. No differences in secondary or tertiary structures were observed in an mAb with 17% glycation [9], but glycation did increase the propensity towards aggregation at accelerated conditions [87]. Several studies have demonstrated that glycation from 10% to close to 100% has no impact on antigen binding [9,47,48], while other studies reported decreased binding affinity caused by glycation in CDRs [37,88]. Highly glycated IgGs have no impact on binding to FcRn, FcγIIIa, and protein A [20], but a slightly decreased binding to FcRn was observed in an acidic fraction containing 17% glycation in another study [9]. The effect of glycation on structure and function may need to be evaluated for each specific mAb because it may vary depending on the location, levels, and specific antigens. Advanced glycation end products can cause coloration of an mAb [89], which may be a concern for product release based on appearance testing. Lys residues with a high propensity towards glycation should be avoided because the use of sugars cannot be eliminated from cell culture medium and feed.

Asp isomerization is favored by mild or slightly acidic pH values, close to the optimal pH range for the liquid formulation of mAbs. Isomerization of Asp in CDRs has been reported to cause local structural changes in CDRs or proximal regions [80,90] and decreased antigen binding [26,79,90,91,92]. Succinimide as an isomerization intermediate in CDRs is more protected from solvent exposure than the original Asp due to local conformational changes [80]. Increased levels of succinimide in a CDR caused decreased antigen binding as either Asn deamidation intermediate [44,93] or Asp isomerization intermediate [26,58,79,91], but one study demonstrated that succinimide at the light chain CDR1 as an Asp isomerization intermediate has no effect on antigen binding [57].

Cys residues are conserved in IgGs with specific linkage for each IgG subclass. The correctly formed disulfide bonds are critical for structural integrity, stability, and biological functions. Acidic species containing 29% unformed disulfide bonds did not show any difference in secondary and tertiary structure [9]. A low percentage of non-reducible species, most likely thioether, does not cause a difference in secondary and tertiary structures [9]. The presence of non-reducible species and free thiols may cause a slight decrease in FcRn bindingbut has no effect on antigen binding [9]. Free Cys residue increases the formation of covalent aggregates [94,95,96]. Unformed disulfide bonding in the heavy chain variable domain has been reported to show slightly increased [49] or decreased [79] antigen binding, but no impact on CDC [49]. Trisulfide bonds have no effect on thermal stability measured by differential scanning calorimetry of an IgG2 molecule [50] and the potency of two IgG1 molecules [97]. IgG2A showed a less compact structure compared to IgG2B and with higher or equal activity as IgG2B [25]. When extra cysteine residues in the light chain CDR3 were modified by cysteinylation, antigen binding was significantly reduced due to steric hindrance [98].

Oxidation of Met can cause the formation of either acidic or basic species when analyzed by chromatography methods. A number of studies showed that Met oxidation in the variable region including CDRs did not affect mAb conformation [80,99], while other studies showed that Met oxidation could alter local conformational changes resulting in decreased flexibility [90] and thermal stability [60]. Several studies reported that oxidation of Met in CDRs has no effect on antigen binding [7,60,90,99,100]. In contrast, one study reported that Met oxidation in the CDRs decreased antigen binding [60]. These apparently conflicting results suggest the importance of selecting appropriate analytical techniques with sensitivity to detect the subtle difference and the dependence of impact on Met residue positions, levels of oxidation, and the specific interactions between antibodies and antigens. A study also reported that oxidation of light chain Met4 (Kabat amino acid numbering system) that is outside CDRs caused decreased binding [37], likely because this position is important for the canonical conformation of the light chain CDR loops. The two conserved Met residues, Met256 and Met432 (Met252 and Met 428 based on Kabat amino acid numbering system), located in the CH2-CH3 domain interface are highly susceptible to oxidation. Oxidation of these Met residues can cause conformational changes around the CH2 and CH3 interface [80,99,101,102], decreased thermal unfolding mainly of the CH2 domain [80,101], and increased aggregation propensity [101]. A forced degradation study found that increased aggregation caused by Met oxidation was only observed when oxidized samples were incubated at 37 °C, but not at 25 °C at both low and neutral pH [81], suggesting a structural perturbation may only occur at a relatively higher temperature. More importantly, oxidation of these two residues caused decreased binding to FcRn [11,12,99,103,104], protein A [12,103,105], and shorter half-life, when present at substantial levels [11,12]. Fortunately, oxidation of these Met residues is typically present at a low level in mAb drug substance and drug product. Oxidation of these two residues has no or minimal effects on binding of a panel of Fcγ receptors [104] but can cause loss of complement binding and CDC due to interference of IgG oligomerization [99].

Various modifications of N-termini are not expected to have a significant effect due to their distance from the areas that are critical to mAb structure and function. However, this theoretical assumption has been contradicted by experimental data. Basic species containing 15% leader sequence did not show any difference in secondary and tertiary structures [9]. However, a basic fraction enriched to 63% of a species retaining the N-terminal leader sequence showed a slight decrease in potency [64], but a different study shows that leader sequence has no impact on antigen binding [9,27]. It is worth mentioning that the lack of detectable difference by antigen binding or other functional assays could also be due to the limited sensitivity of these types of assays. The presence of a nonhuman leader sequence has the potential of causing immunogenicity, especially for repeated administrations. Other examples of N-terminal modifications are the case of an antibody variant lacking two heavy chain amino acids that showed increased affinity to its specific antigen [106]. In addition, cyclization of N-terminal Gln has no impact on antigen binding and potency [27,64].

The impact of C-terminal Lys has been extensively studied. C-terminal Lys does not cause differences in structure and unfolding induced by thermal or chemical stresses [9,30,40] and has no impact on antigen binding and potency [9,27,30,39,40,64,66,67] or FcRn binding [9]. C-terminal Lys removal has no impact on stability after thermal stress for one mAb but decreased stability for a different mAb [67]. Removal of C-terminal Lys may be necessary for optimized binding to the first component of the complement for maximal complement activation otherwise the positive charges of the C-terminal lysine prevent requisite IgG hexamerization [107]. C-terminal amidation has no impact on antigen binding and effector functions [68].

Several factors should be considered for study design and data interpretation. If the goal is to evaluate the impact of total acidic species or basic species, total acidic and basic fractions should be used to establish the direct relationship between these species, and their impact on structure and function. The relationship between a specific modification and its impact cannot be obtained when acidic and basic species each contain more than one modification. If the goal is to evaluate the impact of a specific modification, fractions enriched in this modification should be used. Specific stress conditions, such as high pH for deamidation [108], can also be used to generate material enriched with one specific modification. When applying this knowledge, the percentage of this specific modification in the acidic or basic species needs to be considered. Sensitivities of assays to evaluate structural and functional impacts and the percentage of specific modifications are two factors that cannot be avoided for data interpretation.

## 4. In Vivo Modifications

### 4.1. Animal Model Studies

Animal models have been used to study in vivo degradation of mAbs to establish in vivo and in vitro relationships of various modifications. Deamidation in the CDRs continues to occur in vivo in mice [16] and cynomolgus monkeys [17,18] with similar kinetics as in vitro incubation, suggesting the dominant factors affecting deamidation are temperature and pH. The rate of in vivo deamidation is independent of the route of administration and there is no difference in the clearance rate among mAbs with either the original Asn or deamidation products Asp or isoAsp [17]. The subtle impact of deamidation on FcRn binding [9] is in agreement with the observation that non-substantial difference in FcRn binding may not be translated to in vivo half-life [109]. Succinimide has been shown to be rapidly hydrolyzed to form Asp and isoAsp in cynomolgus monkeys [93], with similar kinetics to in vitro incubation [44,57,110]. Isomerization of susceptible Asp in CDR also occurs in vivo and in vitro with similar kinetics. [16] An unformed disulfide bond in the heavy chain variable domain was shown to be rapidly reformed within one hour in rats [62]. Trisulfide bonds which are stable in buffers and in rat serum were found to be converted into disulfide bonds after 24 h in rats following intraperitoneal injection [97].

Animal model studies have also been used to study the impact of modifications on pK. Glycation increases clearance in mice [111]. C-terminal Lys removal and amidation have no impact on pK in rats [67]. Substantial levels of oxidation of the conserved Met residues in the Fc region caused faster clearance in transgenic mice expressing human FcRn [11,12]. Mice with a human-like defect in N-Glycolylneuraminic acid (Neu5Gc) synthesis generate antibodies to Neu5Gc after injection with cetuximab and circulating anti-Neu5Gc antibodies can promote drug clearance, [112] demonstrating the immunogenicity potential of Neu5Gc and the negative impact on circulating mAbs.

Lower pI has been shown to result in longer half-life in mice demonstrated using mAbs with identical constant domains, and similar FcRn binding affinity but variable domains of different charges [113]. The impact on pK was detectable with a pI difference as low as 0.2 pH units [113]. On the other hand, no difference in pK parameters was observed between acidic, basic, and main peaks separated from mAbs in rats even with a pI difference of approximately 0.4 pH units [9,10]. One possible explanation for the conflicting results could be that the differences in acidic and basic species compared to the main peak separated from an mAb are much smaller compared to mAb charge variants generated by protein engineering or chemical modifications with regard to molecule structures.

### 4.2. Human Studies

Direct experience from human studies is the most relevant information to evaluate the impact of acidic and basic species on safety and efficacy. The presence of the same types of modifications in endogenous human proteins minimizes the risk of safety concerns. The rates of in vivo degradation in humans are critical for setting specifications [19]. Wider acceptance criteria are more tolerable for those modifications with faster in vivo degradation, whereas tighter acceptance criteria are required for modifications with slower in vivo degradation. Fortunately, many of the common modifications have been successfully modeled in vitro with models that translate to observations from clinical results.

Modifications of mAbs after administration into humans are expected and have been shown to continue to evolve in circulation due to physiological conditions. Deamidation of trastuzumab in heavy chain CDR2 continues to occur in circulation in breast cancer patients [74]. In other studies, deamidation of the “PENNY” peptide followed the same kinetics as in vitro incubation [18,19,74] and as human endogenous IgG [19]. There is no difference in clearance among mAbs with the original Asn and those with deamidation products Asp or isoAsp [19]. The C-terminal Lys residue was found to be rapidly cleaved in vivo with a half-life of about an hour [6]. The level of glycation of mAb increases over time in circulation in humans [20] and the kinetics can be duplicated by in vitro incubation under physiological pH, temperature, and glucose concentration [20]. The level of thioether of mAbs increases in circulation in humans by following the same kinetics as in vitro incubation [21]. Conversion of N-terminal glutamate (Glu) to pyroGlu occurs slowly in humans as predicted from the nature of this reaction and follows similar kinetics as in vitro incubation [22]. IgG2A was converted into IgG2B through IgG2A/B in circulation over time catalyzed by thiol-containing compounds as observed during cell culture [114].

Many of the same modifications identified in mAbs have also been detected in endogenous human IgGs. Human endogenous IgG exists with pyroGlu from both Gln and Glu cyclization [22]. On the other hand, minimal C-terminal Lys [6,115] and C-terminal amidation [115] were detected in human IgGs. Glycation has been found present in human endogenous IgG [20], especially in diabetic patients [116]. Approximately 23% deamidation of the “PENNY” peptide was found in human endogenous IgG, as expected based on deamidation kinetics and the half-life of endogenous IgG [16,19]. Oxidation of the same conserved Met residues in the Fc region was also detected in human endogenous IgG [16]. Human endogenous IgG shows approximately 25% mono-sialylation and less than 5% di-sialylation [117,118,119] in the form of N-Acetylneuraminic acid. Free thiols [96,120], trisulfide bond [97], thioether [21], and IgG2 disulfide bond isoform [24,25] have all been reported in human IgGs.

The presence of the same types of modification in humans indicate that a tolerance mechanism has been established. On the other hand, non-human modifications pose relatively higher risks of immunogenicity, such as in the case of Neu5Gc [121]. However, the extremely low level of Neu5Gc may not be a concern as mAbs expressed in murine cell lines have benefited patients of various diseases.

## 5. Control Strategies

Appropriate control strategies are required to make products with consistent quality to ensure safety and efficacy. This includes control of raw materials, manufacturing process steps, testing strategies, and acceptance criteria for specifications and comparability. During the manufacturing process, cell culture is the most impactful stage on acidic and basic species due to the long duration, exposure to complicated media, physiological pH and temperature, and the presence of enzymes secreted by the host cells. To a much lesser degree, downstream processing also has the potential to impact acidic and basic species. Additionally, appropriate formulation and storage temperatures can minimize degradation during storage.

The industry has moved towards the direction of carrying out a comprehensive developability assessment prior to the initiation of substantial chemistry, manufacturing, and controls (CMC) activities [122,123,124]. The objective of the developability assessment is to select a candidate with minimal biochemical and biophysical liabilities that may cause issues during manufacturing and clinical development in parallel with the desired biological activity. A typical workflow of developability assessment includes in silico evaluation at the sequence level using various computing methods to detect many of the potential liabilities outlined above, followed by the high-throughput analysis of biochemical and biophysical attributes, and forced degradation studies to confirm or reject predicted troublesome properties and reveal any remaining hidden risks [122,124].

Charge variant screening is an integral part of the developability assessment. Various analytical methods can be applied to developability and manufacturability evaluation [122,124]. Among them, capillary isoelectric focusing (cIEF) has the advantage of minimal material requirement, minimal method development, and relatively high throughput. It separates charge variants based on net charge differences. IEX-based methods have also been commonly used, but they may require substantial method development and the throughput is low compared to cIEF. IEX-based methods have the additional advantage to detect modifications without direct impact on charges, for example, Met oxidation and several Cys-related modifications. Online LC-MS allows rapid detection of modifications with substantial molecular weight differences enriched in acidic or basic species [125,126]. LC-MS peptide mapping has been the ultimate method to detect, quantify and determine the nature of modifications [124]. Beyond direct analysis, forced degradation studies can be used to accelerate various degradation pathways to increase the detectability of modifications that may appear during long-term stability studies, but present at low levels in drug substances.

For candidate selection, several criteria should be considered. First, abnormal charge distribution may indicate uncommon modifications. As discussed above, the typical charge distribution contains one major peak and several acidic and basic peaks at relatively lower abundance. Charge distribution with more than one major peak or with distinct and abundant acidic or basic species may indicate uncommon modifications or degradation hot spots or extreme cell culture conditions such as the expression of enzymes. Secondly, abnormally high levels of acidic or basic species may indicate higher propensities towards degradations during cell culture, downstream processing, or storage. Thirdly, higher rates of degradations under forced degradation conditions in vitro may correlate with higher levels of in vivo degradation. Last, but not least, simpler charge profiles have the advantage over complicated charge profiles regarding analytical method development, characterization, and control strategies.

### 5.1. Upstream Control

Nowadays, the Chinese hamster ovary (CHO) cell line is the most used production cell line and should be the first choice to consider for producing mAb therapeutics. For alternative consideration, it should be kept in mind that different host cells have been shown to affect acidic and basic species differentially. The nonhuman modification, Neu5Gc, which contributes to the formation of acidic species, is only found in mAbs expressed in murine cell lines [112,127,128,129]. Different cell lines have been shown to result in mAbs with different charge variants and react differently to medium components [130]. More specifically, different cell lines have shown different levels of C-terminal Lys, probably due to variations in the levels of carboxypeptidase activity [63,131,132].

Different clones could also cause a difference in charge profiles due to performance differences such as growth and nutrient consumption [130]. The levels of C-terminal Lys and C-terminal amidation varied with different clones [133]. Several control strategies to mitigate this variability can be used and additional strategies should also be explored based on scientific knowledge. By cell engineering, the levels of enzymes that modify mAbs can be manipulated and thus affect acidic and basic species. Co-expression of the sialidase catalytic domain with mAbs resulted in lower levels of sialic acid [84]. C-terminal Lys has been shown to remain at substantially high levels when carboxypeptidase D was knocked down or knocked out [134]. C-terminal amidation can be substantially reduced by using small interfering RNA to inhibit the expression of peptidyl glycine α-amidating monooxygenase (PAM) or using a zinc finger nuclease approach to generate a CHO line with PAM knock-out [133].

Once a clonal cell line is generated, upstream processes can have a substantial impact on the formation of acidic and basic species. Enzymatic reactions can only occur during the cell culture stage because these enzymes are likely removed by the protein A chromatography, which is typically the first chromatography step of purification. Occasionally, enzymes could be co-purified with products that can cause degradations of mAbs or formulation components that impact product quality [135]. Non-enzymatic reactions are also favored by the longer duration of cell culture, as well as the physiological pH, agitation, and nutrient components present during that stage of the process.

Cell culture operating parameters such as temperature, pH, duration in the bioreactor, and cell density have a profound impact on charge variants. Control strategies can be established based on the relationship of these parameters with charge variants. Overall, longer duration [136,137,138], higher temperature [137,138,139,140,141,142], and exposure to light [143] increased acidic species. N-terminal Gln cyclization mainly occurs during the cell culture stage and is driven by pH, temperature, and duration [144]. Typically, C-terminal Lys increases with cell culture duration, while C-terminal amidation decreases with the cell culture duration due to the dynamic changes between the amount of mAb products and the respective specific enzymes [115,145]. Lower temperature results in increased levels of basic species caused by incomplete removal of C-terminal Lys due to decreased carboxypeptidase activity [140,141,146]. In one study, trisulfide levels were low on days 12–13, but higher on earlier and later days and varied significantly with bioreactor operating parameters [97]. Higher pH results in lower sialic acid and conversely, lower pH results in higher sialic acid levels [147,148]. IgG2A is produced first and slowly converted into IgG2B through IgG2A/B under physiological conditions and likely catalyzed by the presence of cysteine [114]. Other operation parameters, such as cell density can also affect charge variants [142,149]. The scale could also substantially impact charge variants, which is an aspect requiring careful consideration and close monitoring when scale-up manufacturing.

Studies have demonstrated that cell culture medium, feed components, and feed strategy can affect acidic and basic species. For example, glycation has a high correlation with the amount of sugar in cell culture [47,132,150], and to a lesser degree, an inverse correlation with titer [47,150], which can explain the correlation between higher levels of glucose and higher levels of acidic species [146]. Experiments using medium containing all necessary nutrients for cell growth indicated that high glucose caused a higher level of glycation, while free Lys inhibited glycation [151]. Interestingly, galactose is several-fold more reactive than glucose toward glycation [47]. The level of trisulfide bonds varied with cell densities and feed strategies [97]. Cys levels in the cell culture media and feed is the major factor causing the formation of trisulfide bonds [152]. An aged medium that has been stored for 10 weeks caused increased acidic species [137]. The addition of hydrolysate caused decreased levels of acidic species and increased levels of basic species compared to controls operated at the same temperatures [141].

Supplementing cell culture with specific chemicals can also affect acidic and basic species. Zinc, which is a cofactor of carboxypeptidase, accelerates C-terminal Lys removal, while copper inhibited C-terminal Lys removal, probably due to competition with zinc for the enzyme cofactor binding site [145]. Higher copper concentration caused higher levels of basic species [153], likely due to increased levels of C-terminal amidation as copper is the cofactor of the catalyzing enzyme, PAM [69]. Copper sulfate has been demonstrated to reduce the level of the incomplete variable domain disulfide bond [49,154]. Supplementing cell culture with uridine, manganese, and galactose can also cause a higher level of sialic acid [155,156]. Iron levels correlate with the level of acidic species [130,157]. Supplementation of cell culture media with ascorbic acid or ferric citrate increases the levels of acidic species, while supplementation with bioflavonoids decreases the levels of acidic species [136]. The addition of arginine or lysine to cell culture media can increase the levels of basic species [158], probably by inhibiting carboxypeptidase activity. The addition of antioxidants, either rosmarinic acid, iron, or both decreased acidic species from glycation by modulating oxidative stress [137]. The addition of sodium butyrate in the cell culture medium increases the portion of fully assembled molecules while decreasing acidic species and increasing basic species [159]. Adding human sialic acid to a cell culture medium can reduce the Neu5Gc content of cultured human and nonhuman cell lines and their secreted glycoproteins [112].

Degradation products of cell culture components and cell metabolites can also affect acidic and basic species. Using chemically defined media, it was found that ascorbic acid, a common component of cell culture medium, can form the degradation product, xylosone, which contains reactive carbonyl groups and modifies various amines (e.g., the N-termini of the heavy and light chains and susceptible lysine residues), forming either hemiaminal or Schiff base products [54]. As a degradation product of glucose or lipid or metabolite, methylglyoxal can modify arginine residues to generate acidic species [55].

Overall, cell culture is a long process with many parameters that can affect charge variants. Changes in the later days of the bioreactor run are more impactful due to the accumulation of more products. Appropriate controls at the final stages of the bioreactor run are the most critical ones in producing products with consistent acidic and basic species. Continuous process improvement and process changes are inevitable when advancing a program from the pre-clinical stage to early clinical and late clinical stages and ultimately to process performance qualification that is required for the commercial stage. Maintaining acidic and basic species at levels within the historical range is challenging due to their sensitivity toward changes in process parameters in the final bioreactors. Process robustness including scalability should be considered at an early stage to mitigate risks at a later stage while balancing the speed to IND-enabling and first-in-human studies. While cell culture medium and feed are typically well-defined prior to process performance qualification, a risk assessment is carried out using the failure mode and effect analysis (FMEA) to evaluate process parameters such as pH, temperature, feeding strategy, and duration. Special supplements are only considered for special cases or for investigation purposes. Once high-risk parameters are identified, studies are designed using design of experiments (DoE) to define the operating range that should have minimal impact on product quality, including acidic and basic species.

### 5.2. Down-Stream Control

A typical purification process for mAbs includes protein A capture, IEX chromatography, and several filtration steps. Chromatography steps are designed to remove host cell proteins, aggregates, fragments, residual DNA, endotoxin, and other impurities. Filtration steps are mainly used to remove particulates and viruses. Downstream processes can impact acidic and basic species, although not usually as significantly as upstream processing. As discussed in the earlier section, enzymes should be removed by the commonly used protein A chromatography which minimizes the impact of enzymatic reactions on acidic and basic species. Control strategies can be established based on the understanding of process parameters and their impact on charge variants, e.g., low pH and agitation causing aggregation or long time holding at neutral or slightly basic pH causing deamidation.

In general, modifications that affect the binding affinities of mAbs to chromatography and filters can impact acidic and basic species by the removal of specific species. For example, the oxidation of Met256 and Met432 caused an earlier elution from protein A chromatography [105], which can be exploited to remove oxidized mAb variants. Acidic species caused by sulfonated tyrosine are enriched in the strip fractions of an anion exchange column indicating strong interactions [52], thus such a modification should not impact acidic species in bulk drug substances. While widely used to remove aggregates, cation exchange chromatography (CEX) can be explored to modulate the levels of acidic and basic species [160,161], where acidic species eluted in earlier and basic species eluted later as predicted theoretically and proven experimentally. During fractionation across a CEX column, acidic species and fragments are enriched in earlier fractions, while basic species, including C-terminal Lys, aggregates, and sequence variants with the mutation from Glu to Lys, are enriched in later fractions demonstrating that setting appropriate collection criteria can help control the level of acidic and basic species and eliminate sequence variants [71]. Trisulfide bond variants can be reduced by washing with cysteine during the protein A chromatography step [162]. Whether, enriched in the front or the back of the elution peaks, controlling acidic or basic species by setting collection criteria will have a substantial impact on product yield at a large scale and may only be used as the last option to control charge profiles.

As discussed above, several modifications are catalyzed by environmental factors such as pH, temperature, agitation, exposure to light, and duration. The low pH commonly used for viral inactivation and protein A column elution can significantly affect product quality. Studies demonstrated that low pH exposure caused conformational changes leading to aggregation [163,164,165,166,167]. Thus, when achieving sufficient viral inactivation and protein A chromatography, the lower limit of the pH should be carefully studied and controlled to avoid causing substantial conformational changes. Issues arising from low pH viral inactivation can be discovered and addressed through developability assessment. For example, low pH viral inactivation at pH 3.5 followed by pH neutralization resulted in the precipitation of an mAb, which was attributed to two uncommon amino acids at the VL-VH interface that could be resolved by mutations [168]. The pH ranges used for viral inactivation and protein A elution also favors the accumulation of Asp isomerization intermediate, succinimide [57]. Sample hold times are predicted to affect acidic and basic species by introducing additional modifications, especially for those that do not require harsh conditions or for matrices that are closer to physiological pH. For example, using synthesized peptides, it was found that the hold time and buffer compositions have a substantial impact Gln to pyroGlu conversion rate, whereas buffer pH has less of an impact [144]. Asn deamidation is highly dependent on buffer types, pH, temperature, and duration [169]. Agitation, which is a common stress factor during manufacturing, can cause mAb aggregation [170], which generates either acidic or basic species.

It is desirable to avoid solutions with low or high pH values (such as lower than 3.5 and higher than 8.0), agitation and extended hold time. Charge-based methods are the most sensitive methods to detect subtle changes and should be considered for process intermediate hold time studies. Appropriate control strategies during downstream process should include not to cause an increase in acidic and basic species.

### 5.3. Formulation and Storage

The process of formulation development involves the selection of appropriate buffer components, excipients, surfactants, and pH that will maintain mAb stability during long-term storage by minimizing degradation. Most of the degradations influence the levels of acidic and basic species. N-terminal Gln conversion to pyroGlu is at the minimum at pH 6 and lower temperature [171]. Deamidation of the “PENNY” peptide is well-known to be accelerated at neutral and slightly basic pH, while slightly acidic conditions accelerate deamidation in the VSNK (single amino acid code) peptide [31]. Slightly acidic pH that is commonly used for formulations causes the accumulation of succinimide as an Asp isomerization intermediate [57] and an Asn deamidation intermediate [44]. The rate of glycation in mildly acidic sucrose-containing formulations was proportional to the incubation temperature [172], no glycation was observed at 4 °C even after 18 months, whereas at 37 °C, glycation was observed after just 1 month. Thus, a lower temperature is an efficient way to limit glycation in formulations containing sucrose [173]. Oxidation is accelerated by higher temperatures and exposure to light [174].

Buffer components, excipients, and surfactants have also been demonstrated to cause mAb modifications. Citric acid, a commonly used component to buffer pH has been demonstrated to modify the N-termini of light chain, heavy chain, or both under stressed conditions in mild pH around 5 [53]. At higher temperatures, sucrose hydrolysis was accelerated, resulting in increased levels of glycation and thus aggregation [87], whereas such an impact is minimal at lower temperatures of 2–8 °C [87,172,173]. It is well-known that polysorbates can degrade to form fatty acids and peroxide, which can cause the formation of particulates and mAb oxidation [175,176]. Oxidation of Met residues was observed in formulation buffers containing sodium chloride and polysorbate catalyzed by iron ions due to chloride ion corrosion of stainless steel at low pH [174]. This study further demonstrated that the observed oxidation can be prevented by using formulation buffers without sodium chloride, or without polysorbate, removal of oxygen from the vial headspaces, or the addition of antioxidants.

In general, acidic species and basic species are routinely monitored during stability studies. As discussed in earlier sections, charge-based methods are sensitive to detect subtle changes including chemical degradation such as deamidation and oxidation, and physical degradation such as aggregation. Maintaining consistent levels of acidic and basic species is not as challenging for drug substances because drug substances are typically stored frozen. However, it could be a risk for drug products that are commonly kept at 2–8 °C for at a minimum a year and longer, for most of the cases.

### 5.4. Phase-Appropriate Specification for Acidic and Basic Species

The International Council for Harmonisation of Technical Requirements for Pharmaceuticals for Human Use (ICH) Q6B defines a specification as “a list of tests, references to analytical procedures, and appropriate acceptance criteria which are numerical limits, ranges, or other criteria for the tests described. It establishes the set of criteria to which a drug substance, drug product or materials at other stages of its manufacture should conform to be considered acceptable for its intended use.” It also states that “Specifications are one part of a total control strategy designed to ensure product quality and consistency.”

Specification setting for early-stage programs is typically based on platform specifications and knowledge from similar molecules either internally or from the literature. Molecule-specific characteristics such as biochemical and biophysical properties, mechanism of action, dosing regimen, route of administration, and patient population [177] are also considered for setting specifications. Charge variants have been commonly used as an identification test with acceptable criteria that typically conforms to reference standard. It is recommended to have a specific description in the standard operating procedure to define the pI ranges and peak profiles to provide a clear definition. Charge variants have also been monitored as an important quality attribute to ensure lot-to-lot consistency. Due to limited manufacturing experience, lack of understating of the structure-function relationship, and more importantly lack of direct clinical experience, setting a specification without numerical limits for the relative percentage of acidic, main, and basic, may be acceptable for phase I programs with a rigorous justification based on animal safety data and published information from similar molecules. However, it is preferable, and often requested, to have numerical limits for the main peak, total acidic, and total basic species. In-process testing of the key intermediates to show that the sponsor understands the process and to ensure the process is well-controlled can provide good justification to not set numerical limits or with a wider range for each total acidic, basic, and main species. In deciding whether to set tighter ranges for phase II programs, the direct clinical experience from the phase I study should be seriously considered for specifications and comparability. Late-stage programs are expected to have specifications with numerical limits, justified with manufacturing history with data of in-process, release, stability, and more importantly clinical experience. The specification may be requested for individuals or groups of acidic or basic species to better ensure product safety and efficacy. Further tightening of the numerical limits may be required during the early commercialization phase, for example, after manufacturing 30 batches, based on statistical analysis. The appearance of new peaks or elevated levels of the same peaks in later lots will require thorough characterization to ensure the presence of the same modifications and scientific justification for the lack of impact on safety and efficacy.

Nonhuman modifications should be controlled with tighter specifications for patient safety. Tighter specifications should also be applied to modifications that clear slowly in circulation because the initial levels are the major factor impacting patient exposure, while relatively wider specifications can be applied to attributes that clear quickly in circulation because the difference in the initial level diminishes rapidly in vivo [19].

### 5.5. Critical Quality Attribute Assessment

ICH defines CQA in ICH Q8(R2) as “A physical, chemical, biological or microbiological property or characteristic that should be within an appropriate limit, range, or distribution to ensure the desired product quality”. One scoring system developed and commonly adopted by the industry is based on risk assessment, taking impact and uncertainty into consideration to score each quality attribute and thus categorize quality attributes as either CQA or non-CQA [178,179]. The impact score is evaluated based on biological activity, PK/PD, immunogenicity, and safety with a score ranging from 2 (no impact) to 20 (significant impact). An uncertainty score is evaluated based on the reliability of the information used for evaluating the impact score, where a score range of 1 to 7 is assigned, representing very high to very low confidence. The risk score is derived by multiplying the highest impact score by the uncertainty score. If the risk score is greater than 13, the quality attribute will be assigned as a CQA, otherwise, it is assigned as a non-CQA.

CQA evaluation evolves along with the program development process. For early- stage programs, with no or limited information on the chemical nature of acidic and basic species, lack of structure–function relationship, and clinical experience, acidic and basic species are most likely categorized as CQAs. Although based on the published information, no difference in antigen binding, potency, and pK was observed between acidic, basic, and the main species, [9,10] it may not be justifiable to classify acidic and basic species as non-CQA due to the lack of extended characterization. If not carried out earlier, peak isolation and extended characterization of acidic and basic species will be included in a biologics license application submission. If necessary, the CQA assessment can be re-evaluated. Whether or not classified as a CQA, it is prudent to maintain acidic and basic species at consistent levels. When differences arise, additional peak isolation and characterization are most likely required to demonstrate the presence of the same species and the lack of adverse effects on safety and efficacy. Sometimes, in vitro data alone may not be sufficient to justify the lack of impact, especially for safety.

## 6. Conclusions

Charge variants of therapeutic mAbs have been theoretically and experimentally established as the rule, rather than the exception. Acidic and basic species have drawn substantial attention during the development and commercialization of therapeutic mAbs due to their sensitivity to process changes. It is challenging to maintain the levels of acidic and basic species within the reasonable ranges defined in specification and comparability acceptance criteria when process changes were introduced for process optimization, manufacturing scale-up, and transfer. Candidate selection through developability assessment, early phase process development, and formulation development are critical steps toward successful late-phase development and commercialization. The objective of the developability assessment is to select a candidate with inherent properties of generating low and consistent levels of acidic and basic species. The inclination towards advancing a program through IND-enabling toxicology and early phase development quickly should be balanced with the need to understand the degree of control over charge variants. Since the characterization of the acidic and basic species at the early development stage is not necessary nor is it a common practice, a basic understanding of process parameters and their effects on charge profiles is essential to support process optimization, transfer, and scale-up. Process parameters are further studied, qualified, and tightly controlled during PPQs to be commercialization ready. Overall, parameters during cell culture have the most substantial effect on charge variants. To a much lesser degree, downstream process, formulation, and storage can be explored to control charge variants. The qualitative difference, such as the appearance of new species, is more concerning compared to the quantitative difference. It is more forgiving in early phase development compared to late-phase development and commercialization. Nevertheless, maintaining acidic and basic species within a controlled range throughout development including animal toxicology, early phase, and late-phase development, and commercialization can ensure product safety and efficacy.

## Figures and Tables

**Table 1:** Modifications in acidic species.

Modifications	References
Common	
Asn deamidation	[9,13,14,15,16,17,26,27,28,29,30,31,32,33,34,35,36,37,38,39,40,41,42]
Sialylation	[9,27,30,36,39,43,44,45,46]
Glycation	[9,14,29,34,36,37,39,47,48]
Oxidation	[14,29,30,44]
Cysteine-related modifications	
Unformed disulfide bond	[9,33,39,49]
Non-reducible disulfide bond	[9]
Trisulfide bond	[32,50]
IgG2 disulfide bond isoforms	[24,32,34,50]
Cysteinylation and glutathionylation	[39]
Fragmentation	[9,14,33,39,44,45]
Rare	
Asp more acidic than isoAsp	[26,28]
Succinimide intermediate of Asp isomerization	[34]
IsoAsp from Asp isomerization	[32]
Maleuric acid modification	[51]
Tyrosine sulfation	[52]
Citric acid modification	[53]
Xylosone modification	[54]
Leader sequence	[38]
Sequence variant from Gly to Asp	[29]
Arginine modification by methylglyoxal	[55]
Aggregates	[33]

**Table 2:** Modifications in basic species.

Modifications	References
Common	
C-terminal Lys	[9,13,27,28,30,32,34,36,39,40,43,45,63,64,65,66,67]
C-terminal amidation	[32,34,67,68,69]
Succinimide intermediate	[26,44,57,59]
IsoAsp as Asp isomerization	[16,26,34,42]
Leader sequence	[9,27,32,34,38,45,64,69]
Aggregates	[9,10,33,40]
N-terminal uncyclized Gln	[27,62,64,65]
Oxidation	[36,46,60,61]
IgG2 isoforms	[24,32,34,50]
Rare	
Sequence variant Ser to Arg or Glu to Lys	[70,71]
Unformed disulfide bonds	[33,62]
Cysteinylation	[38]
Cyclization of N-terminal Glu to pyroGlu	[39,69]

## Data Availability

Not applicable.

## Abbreviations

ADCC	Antibody-dependent cellular cytotoxicity
Asn	Asparagine
Asp	Aspartate
BLA	Biologics license application
CDC	Complement-dependent cytotoxicity
CDR	Complementarity-determining region
CHO	Chinese hamster ovary
CEX	Cation exchange chromatography
cIEF	Capillary isoelectric focusing
CMC	Chemistry, manufacturing, and controls
CQA	Critical quality attribute
Cys	Cysteine
DOE	Design of experiment
Fab	Fragment antigen binding
Gln	Glutamine
Glu	Glutamate
ICH	The International Council for Harmonisation of Technical Requirements for Pharmaceuticals for Human Use
IND	Investigational New Drug
IEF	Isoelectric focusing
IEX	Ion exchange chromatography
LC-MS	Liquid chromatography-mass spectrometry
Lys	Lysine
Met	Methionine
NGNA	N-Glycolylneuraminic acid
PAM	Peptidyl glycine α-amidating monooxygenase
PD	Pharmacodynamics
PK	Pharmacokinetics
PPQ	Process performance qualification
PyroGlu	Pyroglutamate
WCX	Weak cation exchange
